# A case presentation of spider lamb syndrome in a Kermanian breed lamb

**Published:** 2015-12-15

**Authors:** Mohammad Naser Nazem, Bahador Shojaei, Akbar Asadi, Mohammad Hasanzadeh

**Affiliations:** 1 *Department of Basic Sciences, Faculty of Veterinary Medicine, Shahid Bahonar University of Kerman, Kerman, Iran;*; 2 *Department of Basic Sciences, Faculty of Veterinary Medicine, Islamic Azad University, Shahre Babak Branch, Kerman, Iran.*

**Keywords:** Inherited abnormality, Lamb, Spider lamb syndrome

## Abstract

Skeletal abnormalities are most often used to describe defects in the arms or legs that are associated with genes or chromosomes, or that occur due to an event that happens during pregnancy. Spider lamb syndrome (SLS) is a congenital disorder in sheep breeding that is recognized by some deformities in skeletal system especially in the limbs. A dead day-old cross-breed white lamb with deformed limbs was referred to the anatomy hall of the Veterinary Faculty of Shahid Bahonar University of Kerman. In the external examination, the lamb was very skinny and in the facial region, superior brachygnathia with a slight Roman nose were observed. Metacarpal and metatarsal regions were more elongated than that expected. Also Metacarpal and metatarsal bones were as long as the antebrachial and crural regions, respectively. This paper, the first report of this syndrome in Iran, described the anatomic and radiographic features of the skeletal deformities in a day-old dead Kermanian breed lamb.

## Introduction

Skeletal limb abnormalities are most often used to describe defects in the arms or legs that are associated with genes or chromosomes, or that occur due to an event that happens during pregnancy. The abnormalities are often present at birth.^1^ One of these disorders in sheep is spider lamb syndrome (SLS),^2^ also known more formally as hereditary chondrodysplasia.^2^^,^^3^ It is a congenital osteopathy first described in young lambs during the early 1970s.^3^ Several skeletal abnormalities are associated with the syndrome, including disproportionately long, spider like legs, curvature of the spine, deformed ribs and sterna, facial deformities, lack of body fat and muscular atrophy.^3^^,^^4^ The most noticeable condition is an outward bending of the forelimbs from the carpal joints, with many lambs also having a crooked spine (mentioned before) in the thoracic area and a marked Roman nose. In addition, these lambs show extreme height, fineness of bone, poor muscling and failure to thrive.^2^ Embryonic development at the ends of the long bones is impaired and lambs end up on the ground with their legs splayed like a spider.^5^

This syndrome was primarily observed in United State black – faced Suffolk and Hampshire sheep.^2^^,^^4^^,^^6^^,^^7^ According to some researches the presence of SLS was originally confined to sheep derived from Suffolk breeding stock, suggesting the mutation most likely arose within this breed.^8^ As mentioned before, it is speculated that the mutation probably occurred in the late 1960s and was disseminated through the use of a popular genetic for its production and show – ring performance. Within the last two decades, the syndrome has been reported in several sheep breeds, presumably due to crossbreeding.^4^^,^^9^ Reduction in the number of viable lambs born per ewe can have a major economic impact on lamb production systems.^4^^,^^10^ Thus, the presence of a congenital disorder such as SLS can have a significant effect on production efficiency. The elimination of SLS from affected sheep population by identifying individuals that are carriers of the mutation would be of benefit to the sheep industry.^4^ The aim of this article was is to describe the anatomical features of a lamb affected with the spider lamb syndrome.

## Case Description

A dead one day-old cross-breed white lamb with deformed limbs was referred to the anatomy hall of the Veterinary faculty of Shahid Bahonar University of Kerman on March 2014. In the physical examinations, the lamb was very skinny and there wasn’t a notable fat layer under the skin. In the facial region, superior brachygnathia with a slight Roman nose was observed. Also there was an ankylosis in the tarsal joint.

**Fig. 1:**
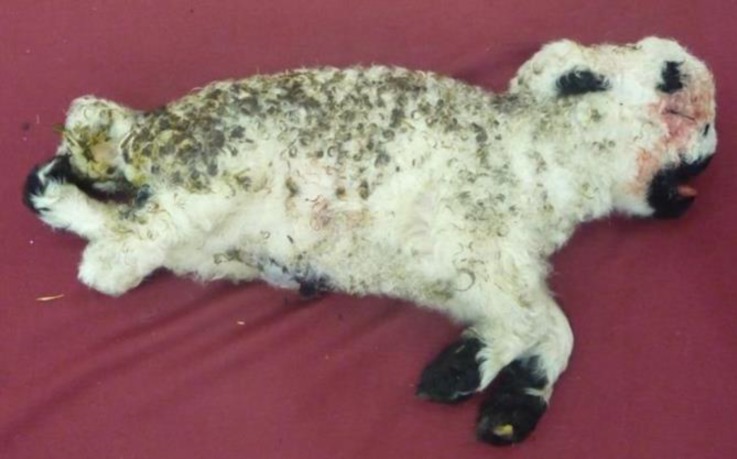
Cross-breed white Kermanian lamb. Superior brachygnathia with a slight Roman nose is observed in the skull. Deformed limbs and mild kyphosis in the thoracic region are also seen

Metacarpal and metatarsal regions were more elongated than that should be expected. Also metacarpal and metatarsal bones were as long as the antebrachial and crural regions, respectively (Fig. 1). The dorsoventral and latero-medial radiographic images were acquired using 60 kVp and 5 mAs with FFD of 80 cm (Xvision EX; Toshiba, Nasu, Japan). Finally in order to reveal the abnormal structures the lamb was dissected carefully. 

Dissection of the forelimb revealed that extensor muscles of the digits were originated normally from the lateral surface of the radius, but their tendons were run at the lateral border of the radius to the carpal joint and finally deviated to the caudolateral aspect of the carpal joint to reach to the metacarpal bone. At the metacarpal region according to the outward bending of the forelimb distal to the carpal joint, extensor tendons run slightly to the cranial surface. It had resulted in the change of dorsopalmar axis of metacarpal to the lateromedial one. According to this change, extensor digital tendons in the metacarpal region were observed on the lateral surface of the metacarpal bone but their divisions were normal proximal to the fetlock. Due to these events, normal lateromedial arrangement of the extensor tendons of the metacarpal region had been changed to the caudocranial position (Fig. 1). The cranial tendon, extensor digitrum communis, was divided into the two lateral and medial branches proximal to the fetlock. Medial branch was continued to the medial digit while lateral one divided to two lateral and medial branches which were run to the lateral and medial digits, respectively. The tendon of caudal muscle, extensor digitrum lateralis, was continued to the lateral digit similar to normal arrangement.

The flexor muscles of the digits were originated from the caudoproximal part of the radius except superficial digital flexor muscle which originated normally from the medial epicondyle of the humerus. According to the radius deviation, flexor tendons were passed to the its medial side. At the carpal region, they were seen on the caudomedial surface. Also superficial digital flexor tendon was situated medially in compared to the deep digital flexor tendon. 

According to our examinations, the axis of carpus was dorsolateral to mediopalmar instead of dorsopalmar. This position caused the carpal canal to displace to the mediopalmar of the forelimb. This malposition had led to move the accessory carpal bone to displace to the mediopalmar surface. 

In the distal joint, the lateral phalanges were deviated to lateral while medial ones turned to the medial surface. Phalanges at the proximal and middle joints were normal. 

**Fig. 2:**
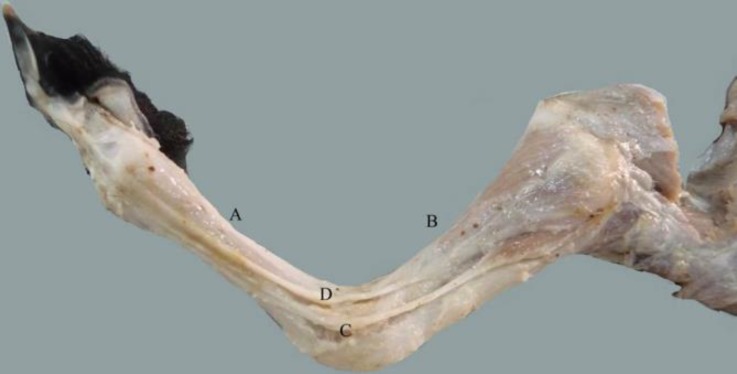
Antebrachial and metacarpal regions. Metacarpal bone (A) is as long as the antebrachial (B). Extensor digitrum communis tendon (C) and extensor digitrum lateralis (D) are shown. Rotation of carpal joint is changed the dorsopalmar axis of this joint

Dissection of the hind limb showed a medial rotation in the tarsus which caused replacement of the calcaneus and associated tendons to the lateral side (Fig. 2). Superficial digital flexor tendon was passed from the medioplantar of the tarsal joint and positioned at the plantar surface of the metatars. 

Deep digital flexor tendon was seen under the superficial one which passed distally to the medioplantar surface of the metatarsal bone. All of the components of the common calcaneal tendon were seen, however with a distolateral direction. It meant that an inward rotation of the hind limbs was taken place.

**Fig. 3:**
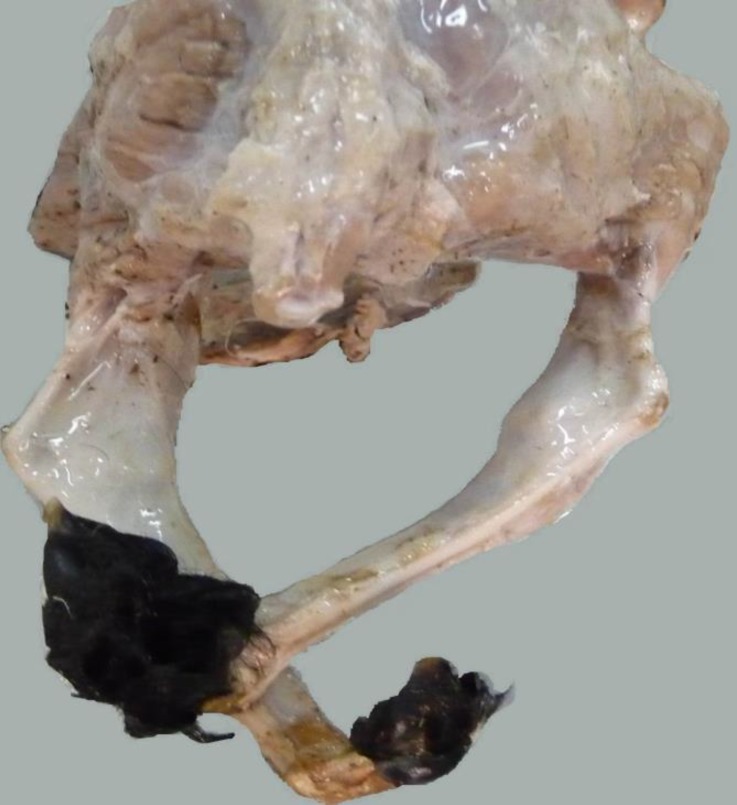
Medial rotation in the tarsal joint that caused replacement of the calcaneus to the lateral side

**Fig. 4:**
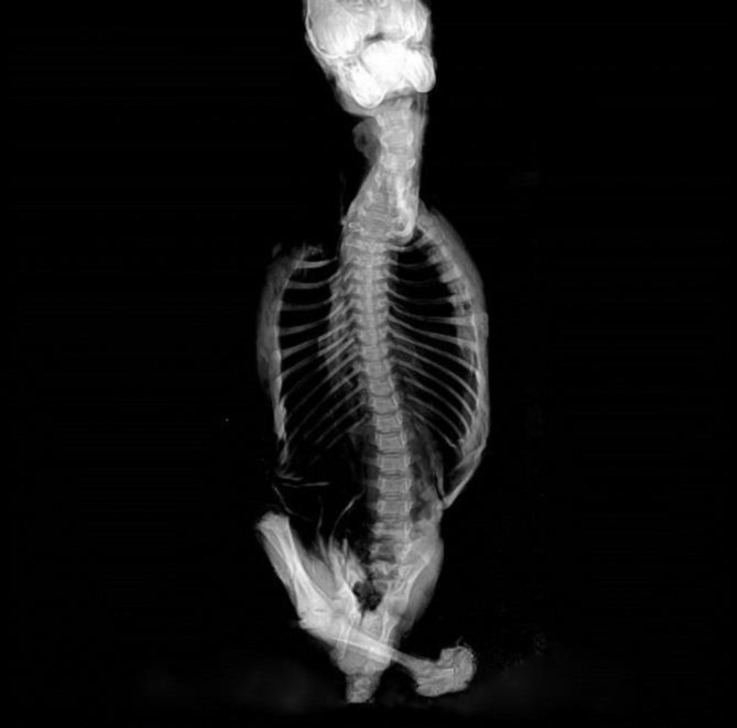
Dorsoventral radiograph shows a scoliosis in the thoracic vertebrae region that continued to the lumbar region

The fetlock joint in the hindlimb showed inward rotation as well (Fig. 3). According to the medial rotation, tendon of extensor digitrum lateralis muscle was displace to the dorsal surface of the tars and lateral to the extensor digitrum commonis tendon. These tendons were positioned normally proximal to the fetlock. Radiographic examination of the vertebral column in dorsoventral view showed scoliosis in the thoracic region (Fig. 4). 

**Fig. 5:**
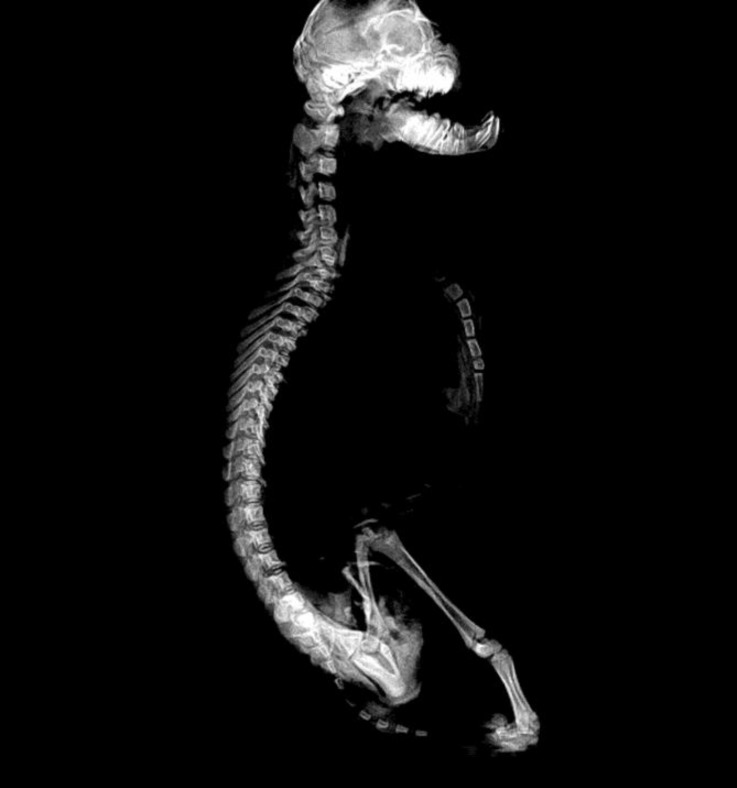
Lateromedial radiograph shows superior brachygnathia, thoracic vertebra spinous process with more caudally oriented with no anticlinal vertebrae and a mild kyphosis

Lateral radiographs of the skull confirmed superior brachygnathia (Fig. 5).

In the lateromedial view of the thoracic vertebrae, spinous processes of thoracic vertebra were observed more caudally oriented (Fig. 5). Also spines in all thoracic vertebrae were inclined caudally. Anticlinal vertebra wasn’t seen (Fig. 5). Radiography of the trunk showed a mild kyphosis in the thoracic region and a concavity in the sternum in this view (Fig. 5).

**Fig. 6:**
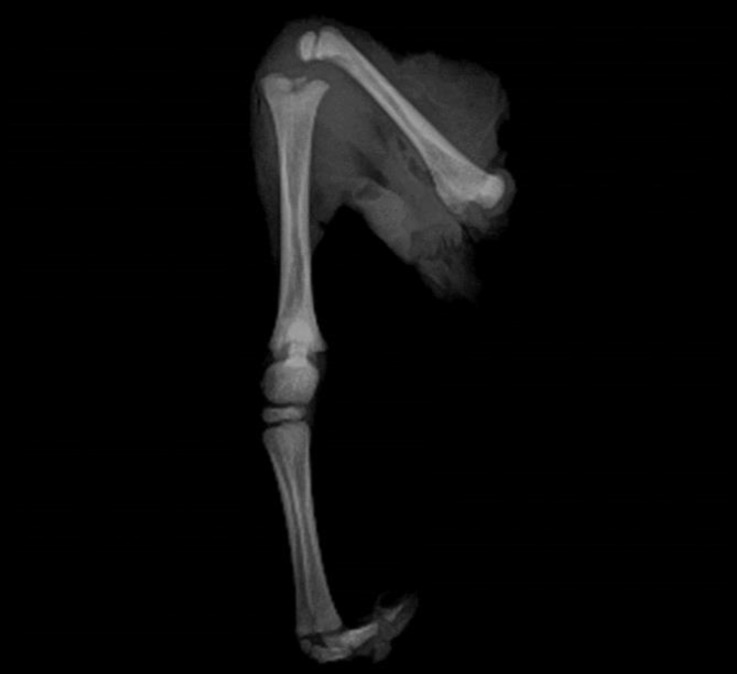
Hind limb radiograph. Absence of growth plate in all bones is seen. The fusion of the calcaneus, talus and central tarsal bones are visible.

The assessment of the total length of the scapula, distal extremity of the humerus, distal extremity of the radius, total length of the ulna, proximal end of the metacarp, proximal extremity of the femur, total length of the tibia and metatars did not show any growth plate (Fig. 5). Radiographs of the tarsal joint showed the fusion of the calcaneus, talus and central tarsal bones. This fusion was also occurred in the distal row of tarsal bones (Fig. 6).

## Discussion

Spider lamb syndrome is characterized by generalized chondrodysplasia and is apparently a semi lethal auto-somal recessive trait. Variable expressivity of the trait may occur in the homozygous animals.^2^ Within the last two decades, the syndrome has been reported in several sheep breeds, presumably due to crossbreeding. Included breeds are North American Suffolks and Hampshires,^6^^,^^9^ and more recently U.S. Southdowns, Shropshires, and Oxfords. In addition, there are reported cases of SLS in Australia and New Zealand following exportation of several U.S. Suffolk sheep to Australia in 1987.^4^^,^^11^ The syndrome has not been reported in Iran. Arachnomelia (arachno = spider and melia = limb) is other name of SLS. Inherited arachnomelia or inherited chondrodysplasia reported by batavani *et al.*, in Iran, previously.^12^ Also, non-carrier identification of spider lamb syndrome in Iranian Baluchi and Karakul sheep has been reported by Nassiry *et al.*^13^ The lamb of this study was a cross breed of Kermanian. 

The lamb in this report was dead at the birth and had some deformities which some of them have been mentioned in references. According to Rook *et al*.,^14^ clinical presentation of the spider lamb syndrome is highly variable, while some lambs were severely affected at birth. The condition develops in the others at 3 to 4 or 3 to 8 weeks of age.^4^^,^^13^ It is characterized by overall appendicular and axial deformities, including kyphosis, scoliosis, concavity of the sternum, lateroventral deviation of the maxilla (crooked nose and Roman nose), and angular deformities.^2^ According to our findings, this lamb had long bent bones in its limbs especially in the zeugo and autopodium. Because of abnormal asymmetrical growth, the positions of the tendons in these regions, the lamb was showed varus/ valgus abnormality. It means that a non-symmetrical growth has been taken place in affected bones.

One method to recognize the spider lamb syndrome is the evaluation of the radiographic changes. Radiological evaluation of the shoulders, elbows, and sternum from affected lambs reveals multiple irregular islands of ossification and the sternebrae appear irregularly in sized and symmetry.^2^^,^^8^^,^^14^ The most constant radiographic sign is in the olecranon, which exhibits multiple islands of ossification instead of the uniform, nonmineralized cartilage surrounded by dense bone in a normal lamb.^2^ In the radiographic assessments, there were not growth plates in some appendicular bones which is the first report on this syndrome. Also we could not observed islands of ossification in the fore and hind limb in radiographs. It is in agreement with Smith that stillborn lambs or those that died in the first week of life did not exhibit radiographic abnormalities in growth plates associated with spider syndrome.^2^

Some abnormalities of the skeletal system of the current case such as scoliosis, mild kyphosis, concavity of the sternum, long bent limbs and angular deformities have been reported by other researchers^2^^-^^4^ while some of our observations such as deformities in spinous process of vertebrae, brachygnathia superior absence of the anticlinal vertebra, lack of growth plate in some appendicular bones and ankylosis of tarsal bones have not been recorded previously. In the skull the dorsal surface of the nose was dome shaped denoted as Roman nose.^2^

Sheep have 54 chromosomes, with 26 pairs of auto-somes and two sex chromosomes.^3^ Many researchers have mapped the spider lamb syndrome locus to the distal end of ovine chromosome.^3^^,^^7^ Comparative analysis of genome maps between sheep, cattle and humans, combined with the results of knockout studies in mice, has identified fibroblast growth factor receptor 3 (FGFR3) as a positional candidate for the disorder.^3^ Lambs homozygous for the SLS allele (FGFR3 SLS/SLS) have bone deformities. In contrast, heterozygous lambs (FGFR3SLS**/**+) appear normal, though perhaps through relaxed inhibition of chondrocyte proliferation at the growth plate, are physically larger than normal lambs.^15^^,^^16^ Smith *et al.* and Beever *et al.* proposed that FGFR3SLS/+ sheep will exhibit enhanced long bone growth and greater frame sizes at all ages, which translates to greater BW and a larger LM area.^16^^,^^17^

The FGFR3 loss-of-function mutations (i.e, SLS) cause skeletal overgrowth.^17^ It can be an appropriate response to increase the height of the appendicular bones such as this case. 

In order to control this SLS, all the carrier breeders must be actively omitted from the flocks. Traditional breeding methods such as progeny testing of potential breeding rams would reduce the frequency; however, they are time consuming and unaffordable strategies. Thus, the identification of a genetic marker for SLS would be of benefit for monitoring or eliminating it from affected sheep populations. Additionally, the chromosomal localization of the SLS locus is the first step toward positional cloning of the gene responsible for the syndrome.^2^
